# Case Report: Unlocking opportunities in HER2-targeted antibody-drug conjugates for bulky leptomeningeal metastatic breast cancer

**DOI:** 10.3389/fonc.2025.1559085

**Published:** 2025-08-13

**Authors:** Alessandro Leal, Douglas Kondziolka, Donato Pacione, Stacy Antwi, Sylvia Kurz, Nancy Lin, Sylvia Adams

**Affiliations:** ^1^ NYU Grossman School of Medicine, New York, NY, United States; ^2^ NYU Langone Health, Perlmutter Cancer Center, New York, NY, United States; ^3^ Department of Neurosurgery, NYU Langone Health, New York, NY, United States; ^4^ Department of Neurology, Smilow Cancer Hospital, Yale Cancer Center, New Haven, CT, United States; ^5^ Department of Medical Oncology, Dana-Farber Cancer Institute, Boston, MA, United States

**Keywords:** metastatic breast cancer, leptomeningeal disease, HER2-targeted therapy, antibody-drug conjugate, trastuzumab deruxtecan

## Abstract

Leptomeningeal carcinomatosis (LC) is a severe complication of metastatic breast cancer (mBC), with rising incidence. The prognosis for patients with LC has been poor, with a median overall survival of approximately four months. However, recent therapeutic advances, in particular the introduction of trastuzumab deruxtecan have dramatically changed the landscape of CNS metastases and improved outcomes. Here, we present the case of a 42-year-old woman with recurrent HER2+ breast cancer who developed extensive LC after multiple lines of treatment. Despite progressive disease, the patient exhibited a sustained response to trastuzumab deruxtecan, a novel antibody-drug conjugate (ADC), for 15 months, which was further extended by adding tucatinib. This case underscores the potential of ADCs, like trastuzumab deruxtecan, in controlling both brain metastases and leptomeningeal disease, offering hope for prolonged survival in patients with aggressive HER2+ mBC. Additionally, we highlight the evolving role of clinical trials, molecular profiling, and interdisciplinary care in managing this challenging condition. Ongoing trials continue to investigate new therapeutic options for HER2+ mBC with CNS involvement, promising to further improve outcomes and quality of life for patients facing this devastating disease.

## 1 Introduction

The incidence of leptomeningeal carcinomatosis (LC) has shown an upward trend, particularly in patients with metastatic breast cancer (mBC), likely due to improved survival outcomes from advancements in systemic therapies (1). For some women, progression in the central nervous system has emerged as the primary factor limiting survival (2). Among mBC subtypes, those with HER2-positive (HER2+) tumors are at a notably higher risk for central nervous system (CNS) involvement (3, 4); brain metastases typically retain HER-2 expression (5). In the adjuvant KATHERINE trial (comparing trastuzumab emtansine versus trastuzumab) enrolling patients who had residual disease after neoadjuvant HER2-directed therapy, the incidence of brain metastasis (BM) was approximately five percent in both arms (6). Current treatment guidelines for HER2-positive breast cancer with CNS involvement vary depending on extracranial disease (ECD) status and concordance between primary and brain (7).

**Figure 1:**
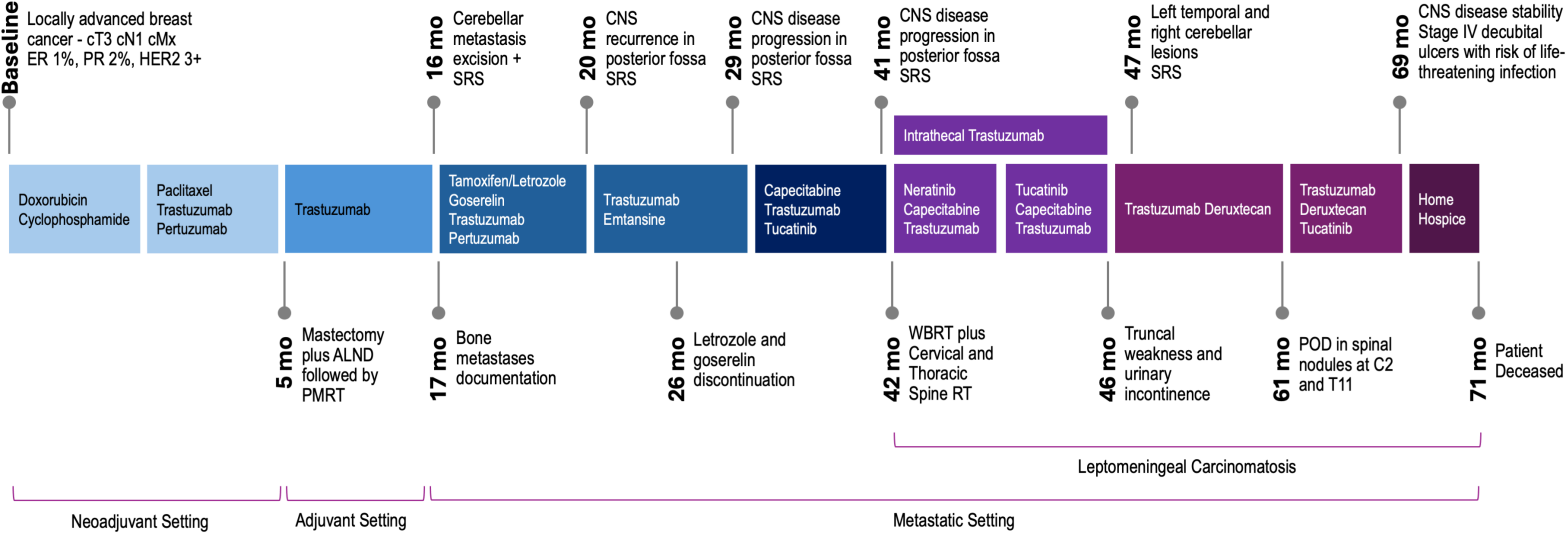
Clinical course and treatment approaches for recurrent HER2 breast cancer with leptomeningeal involvement. ER, estrogen receptor; PR, progesterone receptor; ALND, axillary lymph node dissection; PMRT, post-mastectomy radiation therapy; SRS, stereotactic radiosurgery; POD, progression of disease.

The management of BM and/or LC is tailored based on individual patient characteristics, including performance status, age, control of the primary tumor, and the extent of extracranial disease. For patients with favorable prognostic factors – such as good performance status, age below 65, controlled primary tumor, and limited or absent extracranial metastases – intensive local treatments like surgery, whole-brain radiotherapy, or stereotactic radiosurgery are more frequently recommended (8, 9). Available systemic therapies include biologics such as HER2-directed antibodies (trastuzumab), HER2/3-directed antibodies (pertuzumab), small molecule HER2-directed tyrosine kinase inhibitors (lapatinib, neratinib, tucatinib), several chemotherapeutics (especially taxanes and capecitabine) as well as HER2-directed ADCs (trastuzumab emtansine and trastuzumab deruxtecan). The goals of systemic therapy in patients with intracranial disease, including LC, are to prevent further extracranial progression, enhance intracranial disease control, prevent or palliate neurological symptoms to maintain functional status, and prolong survival. An exploratory analysis of the HER2CLIMB study, which randomized patients to receive tucatinib versus placebo in combination with capecitabine and trastuzumab in patients with advanced HER-2 positive breast cancer, showed that systemic treatment with tucatinib in combination with trastuzumab and capecitabine improved intracranial response rates versus control (47.3% and 20.0%, respectively), in addition to extended overall survival (10). Over recent years, we have witnessed the flourishing of ADCs as a new class of anticancer therapy with enormous capability to improve survival outcomes in patients with metastatic breast cancer, even in heavily treated individuals (11–13). A pooled analysis conducted to evaluate the effectiveness of trastuzumab deruxtecan in the HER2-positive population with BM supported its intracranial activity with an overall response rate (ORR) of 68% in patients with active brain lesions (14). In the DESTINY-Breast03 study, confirmed intracranial ORR was 65.7% with trastuzumab deruxtecan versus 34.3% with trastuzumab emtansine (15). However, none of the studies included in the pooled analysis allowed patients with LC. Here, we present a case of recurrent HER2-positive breast cancer with extensive LC, demonstrating a sustained response to trastuzumab deruxtecan after multiple lines of anti-HER2 therapy (**Figure 1**).

## 2 Case report

A 42-year-old premenopausal woman with no significant past medical history or family history of cancer initially presented with a palpable lump on the right breast. She had recently completed breast feeding for her second child, which was conceived after *in vitro* fertilization. After diagnostic workup, pathology revealed a poorly differentiated invasive ductal carcinoma with lymphovascular invasion, ER low positive (1%), PR low positive (2%), HER2-positive (3+), Ki67 65% and positive axillary lymph node. The patient underwent neoadjuvant chemotherapy with doxorubicin and cyclophosphamide followed by paclitaxel, trastuzumab, and pertuzumab, followed by mastectomy with axillary lymph node dissection which report revealed no residual *in situ* or invasive carcinoma in breast or axillary lymph nodes (ypT0 ypN0), consistent with pathologic complete response. She completed post-mastectomy radiation therapy and adjuvant trastuzumab but declined hormonal therapy due to low hormone receptor positivity. Germline genetic testing was negative for pathogenic variants.

Sixteen months after diagnosis, she presented in a follow up visit with headaches and paresthesia. A brain MRI revealed a 3-cm left cerebellar mass with edema, a craniotomy with lesion excision confirmed metastatic carcinoma consistent with breast origin (GATA3 positive, ER 11-50%, PR-negative, HER2-positive (3+)). Somatic tissue sequencing using a clinical grade panel detected *TP53* D281E, *ERBB3* V104M, *CREBBP* S807N, *ERCC4* I729T, *GALNT12* G272W, *JAK1* Y41C, and *ERBB2* amplification, with a tumor mutation burden of 6 mutations/Mb. She underwent stereotactic gamma knife radiosurgery (SRS) to the tumor bed and further staging workup revealed sustained low burden bone metastases. She started tamoxifen, goserelin, trastuzumab, and pertuzumab and a subsequent bone scan 3 months after first-line initiation showed interval improvement. At the same time, a brain MRI revealed two new dural-based lesions adjacent to the cerebellar resection cavity, CSF cytology was negative, this was followed by SRS to these lesions and initiation of trastuzumab emtansine. A nonspecific CNS lesion was retrospectively noted and subsequently grew to 8-9 mm in the left cerebellum after 8 months on trastuzumab emtansine, she was again treated with SRS.

**Figure 2:**
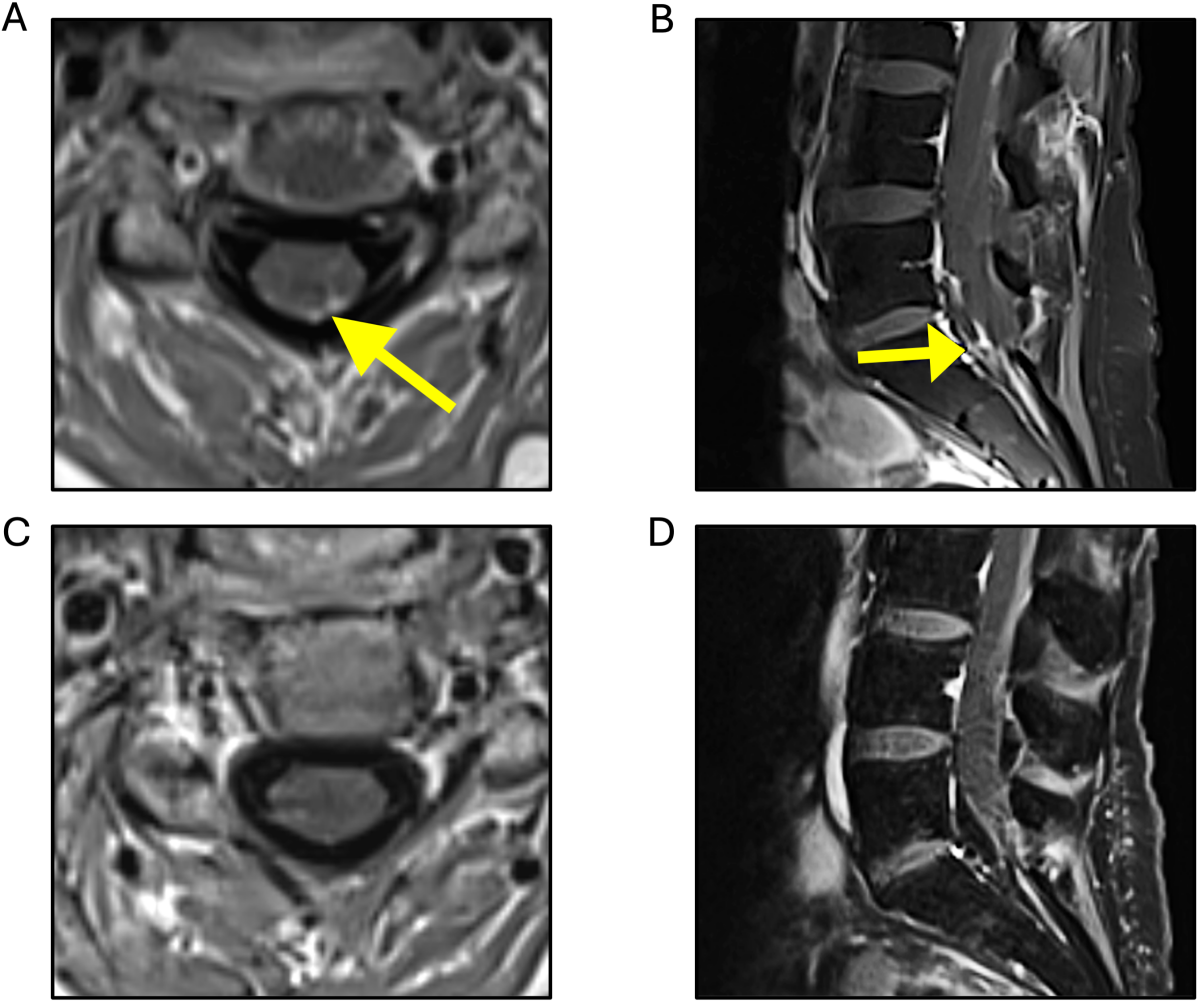
T1-weighted MRI sequences. Multiple areas of nodular enhancement detected, predominantly concentrated along the lower cervical **(A)** and the left-sided cauda equina nerve roots **(B)**, with decreased enhancement observed 2 months after trastuzumab deruxtecan initiation (**C, D**, respectively).

The patient then received tucatinib (unblinded at disease progression), trastuzumab, and capecitabine in the HER2CLIMB trial for 10 months, when serial imaging showed slight worsening of posterior fossa lesions, and she was re-treated with SRS. Shortly after, a brain MRI revealed dural deposits at the left cranial nerve V root extending to the left temporal uncus and hippocampus, concerning for leptomeningeal disease. A spine MRI showed multifocal nodular leptomeningeal deposits and larger lesions at C6 and T2 (**Figure 2**). Whole-brain radiation therapy (WBRT) was initiated, along with targeted spinal radiation. An Ommaya reservoir was placed, and she began intrathecal trastuzumab, along with oral neratinib and capecitabine plus intravenous trastuzumab. Repeated CSF cytology tests were negative. Approximately 5 months after, the patient experienced lower extremity weakness and urinary incontinence, and intrathecal trastuzumab was discontinued due to progression, with new left temporal and right cerebellar lesions treated with SRS. She then started trastuzumab deruxtecan, noting gradual lower extremity strength improvement and increased muscle mass. Five months after trastuzumab deruxtecan initiation, a spine MRI revealed significant improvement of leptomeningeal deposits. Increased edema surrounding the cerebellar lesion resolved with low dose dexamethasone. A subsequently obtained FDG-PET demonstrated low uptake in this area, therefore attributed to prior radiation.

After 12 months of trastuzumab deruxtecan therapy, the patient reported increased left lower extremity weakness. A brain MRI showed no significant changes, but the spinal imaging revealed multiple segmental abnormalities suggestive of a demyelinating process, along with possible new lesions at C2-C4 and T2. Immunofixation showed oligoclonal bands in both CSF and serum, consistent with an autoimmune phenomenon. Three months later, she reported worsening blurry vision, headaches and paresthesia. Brain MRI revealed stable findings, but spine MRI showed disease progression in spinal nodules at C2 and T11. Due to preliminary safety of the combination (personal communication, Dr. Lin), the patient restarted tucatinib, in combination with trastuzumab deruxtecan. After 8 months on this combined regimen, both brain and spine MRIs still showed disease stability, but given the patient’s declining functional status and stage IV decubital ulcers which posed significant risk of life-threatening infection with continued systemic therapy, she was offered comfort care and passed away a few weeks later.

## 3 Leptomeningeal carcinomatosis in HER2-positive mBC

Leptomeningeal carcinomatosis occurs as an advanced-stage complication in approximately 5% of patients with mBC, coinciding with BM in 14% of cases (16–18). While HER-2-positive disease has a higher likelihood of cerebral metastases, this association is not consistently observed for LC. In addition, survival for those with LM, has been poor regardless of subtype, with a median overall survival of approximately 4 months (19). Survival in LC directly correlates with younger age, better performance status, and the ability to receive appropriate treatment, including locoregional, intrathecal, and systemic therapies (20, 21). Furthermore, early access to novel therapies through participation in clinical trials and optimized care through integrated interdisciplinary management, both of which we highlight in our patient, can extend survival. Remarkably, despite bulky LM, and extensive prior systemic therapy, the patient had a sustained 15-month response to trastuzumab deruxtecan, which was then extended by 8 months with the addition of tucatinib.

The challenges of obtaining leptomeningeal tissue samples as well as the low cell count in CSF contribute to the paucity of studies addressing genomic analysis in breast cancer patients with LC. As an alternative, recent studies have shown that molecular profiling of LC can be achieved through circulating tumor DNA sequencing from CSF (22–24). This approach not only aids in diagnosing LC but can also serve as a quantitative marker for monitoring therapeutic responses. In the case presented, genomic analysis of the CSF was not feasible. However, sequencing of brain metastasis tissue obtained at recurrence identified a mutation profile typical of HER2-positive breast cancer.

DESTINY-Breast12 has demonstrated significant BM activity with trastuzumab deruxtecan; within the active BM subgroup, CNS ORR was reported in 19 of 23 patients (82.6%) and in 19 of 38 patients (50%) with untreated and previously treated/progressing BM, respectively (25). The effectiveness of trastuzumab deruxtecan for LC has been studied prospectively, and preliminary data from the LC cohort of the phase II DEBRAH trial (n=7) are promising, with a median overall survival of 13.3 months and 16-week and 24-week overall survival rates of 86% and 71%, respectively (26).

Several ongoing clinical trials expand treatment options for LM in HER2-positive mBC (**Table 1**), with promising early data from a radiotherapeutic trial with intrathecal Rhenium (186Re) obisbemeda (27). Importantly, two phase 3 clinical trials – CompassHER2 RD and DESTINY-Breast05 have been designed to explore the efficacy of newer agents for preventing relapse, including in the CNS, in high-risk HER2-positive breast cancer with residual disease after neoadjuvant HER2-directed therapy (**Table 1**).

**Table 1:** Ongoing clinical trials for leptomeningeal carcinomatosis treatment (top) or CNS disease prevention (bottom) in HER2-positive breast cancer.

NCT Number	Study Description	Study Phase	Interventions	Estimated Enrollment	Primary Outcome
NCT03696030	HER2-CAR T Cells in Treating Patients with Recurrent Brain or Leptomeningeal Metastases	Phase 1	Chimeric Antigen Receptor T-Cell Therapy	39	Incidence of dose limiting toxicities, treatment related adverse events
NCT04588545	Radiation Therapy Followed by Intrathecal Trastuzumab/Pertuzumab in HER2+ Breast Leptomeningeal Disease	Phase 1/Phase 2	Pertuzumab, Trastuzumab, Radiation	39	Maximum tolerated dose, 1-year overall survival
NCT06016387	Tucatinib With Brain and/or Spinal XRT in Patients with HER2+ Metastatic Breast Cancer and LMD	Phase 2	Tucatinib, Trastuzumab, Capecitabine, and Brain & Spinal Radiation	30	Overall survival
NCT05800275	Capecitabine, Tucatinib, and Intrathecal Trastuzumab for Breast Cancer Patients with Leptomeningeal Disease	Phase 2	Tucatinib, Trastuzumab, Capecitabine	30	Overall survival rate at 12 months
NCT04457596	T-DM1 and Tucatinib Compared With T-DM1 Alone in Preventing Relapses in People with High Risk HER2-Positive Breast Cancer, the CompassHER2 RD Trial	Phase 3	Trastuzumab Emtansine plus Tucatinib vs. Trastuzumab Emtansine	1031	Invasive disease-free survival
NCT04622319	Trastuzumab Deruxtecan Versus Trastuzumab Emtansine in High-risk HER2-positive Participants with Residual Invasive Breast Cancer Following Neoadjuvant Therapy (DESTINY-Breast05)	Phase 3	Trastuzumab Deruxtecan vs. Trastuzumab Emtansine	1600	Invasive disease-free survival

## 4 Final considerations

The management of LC in HER2-positive mBC remains a highly complex clinical challenge, however, the introduction of antibody-drug conjugates such as trastuzumab deruxtecan, with accumulating data showing prolonged intracranial and leptomeningeal disease control gives hope for extending survival and improving quality of life for patients with this aggressive disease. Furthermore, investigation of ADCs in patients with early, high-risk breast cancer will hopefully lessen recurrence risk and the burden metastatic disease puts on patients and their families.

## Data Availability

The original contributions presented in the study are included in the article/supplementary material. Further inquiries can be directed to the corresponding author.
